# Silkworm and Silk: Traditional and Innovative Applications

**DOI:** 10.3390/insects13111016

**Published:** 2022-11-03

**Authors:** Silvia Cappellozza, Morena Casartelli, Federica Sandrelli, Alessio Saviane, Gianluca Tettamanti

**Affiliations:** 1 Council for Agricultural Research and Economics, Research Centre Agriculture and Environment, Sericulture Laboratory, 35143 Padova, Italy; 2 Department of Biosciences, University of Milan, 20133 Milano, Italy; 3 Interuniversity Center for Studies on Bioinspired Agro-Environmental Technology (BAT Center), University of Napoli Federico II, 80055 Portici, Italy; 4 Department of Biology, University of Padova, 35131 Padova, Italy; 5 Department of Biotechnology and Life Sciences, University of Insubria, 21100 Varese, Italy

The various subjects covered in the present Special Issue “Silkworm and Silk: Traditional and Innovative Applications” demonstrate how sericulture, a practice deeply rooted in human history, can act as a bridge to bring together an exceptionally wide range of scientific and technical expertise in both conventional topics and cutting-edge technologies.

As with many other species, *Bombyx mori* was obtained through human-driven selection from a wild ancestor but, in contrast to other animals, the genetic stocks of silkworm strains have been preserved in public research facilities for centuries. Therefore, in nations with a history of sericulture, there are wide collections of various genotypes that must be maintained through a customary activity. Additionally, the majority of sericultural regions in the world continue to practice agricultural production of cocoons through conventional silkworm rearing. Since the mulberry leaf is the only food source for the larvae of this insect (or an artificial diet based on it), there has always been a need for moriculture, and the same organizations dedicated to maintaining *B. mori* genetic resources typically preserve mulberry germplasm collections as well; a significant amount of research is devoted to understanding the traits of these varieties and how they relate to the silkworm. A multilevel study on the effects of regional mulberry genotypes on the health of the silkworm is reported in this issue; the authors demonstrated that the selection of mulberry varieties characterized by specific qualitative and quantitative features can strongly impact larval development, cocoon production, and raw silk parameters [1]. Scientists also learnt how to use mulberry varieties to feed other animals or as a source of raw materials and active chemicals; further, they adopted new technologies to obtain advanced materials from silk. This issue presents a bibliometric paper by Giora et al. [2], which analyzed recent research trends in the fields of sericulture and moriculture and provides an overview of the past 20 years of research in this sector of rising interest. Two other reviews [3,4] aimed to connect advances in or criticisms of sericulture to societal and economic changes in various regions of the world, highlighting how this activity may be heavily dependent not only on technological improvements, but also on many external factors.

One of the newest trends in sericulture is to promote the development of circular economy supply chains to employ silk by-products for feed and food, a topic that is comprehensively discussed and documented in the article by Tassoni et al. [5]. Nowadays, silk by-products are easily exploitable via melting silk with the right solvents to obtain solutions of silk proteins, opening the door to entirely new applications in industry including in the textile sector, which is still of the utmost importance for an economy dependent on traditional sericultural agroindustry. Furthermore, this issue contains numerous articles that address these novel applications and cover both the general role of silk fibroin in biomedicine [6] and methods for creating hybrid materials, for instance, by adding nanoparticles [7]. The paper by Bassani et al. [8] examined particular devices, such as silk nerve conduits, in a study case illustrating how research findings can be translated into medical products for use in clinical settings.

Advances in methodology and knowledge are not limited to the new biotechnological uses of silk proteins. A significant part of the Special Issue is devoted to papers using the most recent techniques to study silkworm physiology to enhance silk production. This can improve the general knowledge of silkworm biology and hasten the potential use of this organism in various applied fields. Specifically, Yokoi et al. [9] undertook an RNA-seq investigation of several silkworm larvae tissues, including the midgut, fat body, testis, ovary, and the different subregions of the silk gland, generating reference transcriptome data for a *B. mori* strain. Masuoka et al. [10] identified novel silk protein regulation factors, using a proprietary software, to conduct co-expression networks and time-course expression analyses on transcriptomic data from various tissues, including different subregions of the silk glands, in reference silkworm strains. Zhang et al. [11] performed a transcriptomic analysis of fat body derived from silkworms treated with hydrogen sulfide, a molecule that, at specific doses, exerts beneficial effects on the developmental and economical traits of *B. mori*. All these results provide data and methods with which to develop strategies for enhancing silk production in *B. mori*. Moreover, de la Peña et al. [12] compared RNA-seq transcriptional profiles of silk glands belonging to various silkworm strains commonly reared in the Philippines and maintained under different temperature conditions, producing a set of information useful for the genetic improvement of *B. mori* strains and to increase their silk productivity in this geographical area. Finally, the article by Baci et al. [13] reports a comprehensive overview of the most advanced genome-editing techniques performed in *B. mori,* using the CRISPR/Cas9 methodology. In their paper, the authors illustrated strategies used to obtain both knock-out and knock-in *B. mori* strains. They also focused on the possible applications of CRISPR/Cas9 to generate silkworm strains characterized by a higher resistance to viral infections, highlighting the importance of this technology for both basic research and applied purposes in *B. mori*. Since *B. mori* is considered a model species among Lepidoptera, it has been extensively employed in studies of physiology, genetics, molecular biology, and pathology. Zhang et al. [14] made use of this lepidopteron to investigate how hemolymph ecdysteroid titer affects maternal genes during oogenesis, demonstrating that maternal mRNAs begin accumulating in the ovary during the larval period, at the wandering stage. In the article by Chang et al. [15], the role of polyamines in regulating cell cycle progression and DNA replication was explored as well as the expression of genes coding for enzymes involved in the polyamine pathway in different tissues. Since it is known that the administration of polyamines to *B. mori* larvae increases the expression of the gene coding for fibroin heavy chain and improves the quantity and quality of silk, this study opens the door to manipulating the expression of key genes involved in the polyamine pathway in silk glands to improve silk production. The use of silkworms and insects in general as model organisms began in response to growing concerns about animal health and welfare, with the goal of preventing, or at least reducing, the usage of higher animals in all sectors of the life sciences. In fact, the principles of the 3Rs (Replacement, Reduction, and Refinement), developed in the 1950s, offer a foundation for conducting more compassionate animal research. Since then, they have become an integral part of national and international laws and regulations governing the use of animals in research and are progressively leading to the development of suitable alternative models, among invertebrates, for experimental aims. Holometabolous insects represent a promising option due to their lower costs, convenience in handling, lack of ethical restrictions, and features that can successfully reproduce different biological mechanisms occurring in mammals. In this Special Issue, three articles pave the way for the development of the silkworm as an alternative model for screening antimicrobial compounds against nosocomial pathogens, such as *Staphylococcus epidermidis* [16], and bacteria that cause skin diseases and systemic infection, such as *Cutibacterium acnes* [17], as well as for studying the effects of natural compounds with hypoglycemic activity [18]. Although it is still up for debate whether these insect-based models are applicable in clinical settings, the findings of these three studies are undoubtedly encouraging. Another article focused on the health status of the silkworm and its pathologies. In particular, the study dealt with nuclear polyhedrosis, a major viral disease in sericulture that causes high larval lethality and great economic losses. By using a genomic approach that considered different BmNPV strains, the authors demonstrated that mutation and rearrangement of the genome can lead to differential pathogenicity of this virus [19]. The holobiont concept, which has recently emerged as a theoretical and experimental context in which to study the interactions between hosts and their associated microbial communities, is worth mentioning while discussing the silkworm as a laboratory model. It is becoming increasingly clear that the development, growth, and health of macro-organisms are influenced by the complex microbial communities they host. The article by Dee Tan and Bautista [20] deals with this topic; the authors studied the gut microbial composition of four *B. mori* strains reared in the Philippines to construct a database of useful information fostering an improved understanding of whether and how the gut microbiota affects silk production and to develop strategies for future improvement of the strains. Another topic that has attracted increasing attention in recent years is the administration of probiotics to provide health benefits, generally by improving or restoring the gut microbial community. Unban et al. [21] isolated from the midgut of *Samia ricini*, another lepidopteran species used for silk production, a xylose-utilizing lactic acid bacterium, *Enterococcus hirae* SX2, which acts as a probiotic in this insect due to its tannin tolerance and antimicrobial activity against insect pathogens. The results presented in this article demonstrate that the oral administration of *E. hirae* SX2 to Eri silkworm not only improves its growth and reduces mortality, therefore positively influencing its economic traits, but also represents a starting point for developing strategies to select novel probiotics that can positively affect *B. mori* larvae and improve sericulture research.

Taken together, the articles in this Special Issue cover the transversality of all the topics encompassed by sericulture, which goes far beyond silkworms and silk. This collection of many articles from different countries in a relatively limited period of time testifies to the great interest the scientific community takes in sericulture, encouraging the guest editors to repropose the subject in the near future.;
